# A Deep Learning Approach for Featureless Robust Quality Assessment of Intermittent Atrial Fibrillation Recordings from Portable and Wearable Devices

**DOI:** 10.3390/e22070733

**Published:** 2020-07-01

**Authors:** Álvaro Huerta Herraiz, Arturo Martínez-Rodrigo, Vicente Bertomeu-González, Aurelio Quesada, José J. Rieta, Raúl Alcaraz

**Affiliations:** 1 Research Group in Electronic, Biomedical and Telecommunication Engineering, University of Castilla-La Mancha, 16071 Cuenca, Spain; alvaro.huerta@uclm.es (Á.H.H.); arturo.martinez@uclm.es (A.M.-R.); 2 Clinical Medicine Department, Miguel Hernandez University, 03202 Elche, Spain; vbertomeu@umh.es; 3 Cardiology Department, Hospital General Universitario de Valencia, 46014 Valencia, Spain; quesada_aur@gva.es; 4 BioMIT.org, Electronic Engineering Department, Universitat Politecnica de Valencia, 46022 Valencia, Spain; jjrieta@upv.es

**Keywords:** atrial fibrillation, continuous wavelet transform, convolutional neural network, deep learning, quality assessment, single-lead ECG

## Abstract

Atrial fibrillation (AF) is the most common heart rhythm disturbance in clinical practice. It often starts with asymptomatic and very short episodes, which are extremely difficult to detect without long-term monitoring of the patient’s electrocardiogram (ECG). Although recent portable and wearable devices may become very useful in this context, they often record ECG signals strongly corrupted with noise and artifacts. This impairs automatized ulterior analyses that could only be conducted reliably through a previous stage of automatic identification of high-quality ECG intervals. So far, a variety of techniques for ECG quality assessment have been proposed, but poor performances have been reported on recordings from patients with AF. This work introduces a novel deep learning-based algorithm to robustly identify high-quality ECG segments within the challenging environment of single-lead recordings alternating sinus rhythm, AF episodes and other rhythms. The method is based on the high learning capability of a convolutional neural network, which has been trained with 2-D images obtained when turning ECG signals into wavelet scalograms. For its validation, almost 100,000 ECG segments from three different databases have been analyzed during 500 learning-testing iterations, thus involving more than 320,000 ECGs analyzed in total. The obtained results have revealed a discriminant ability to detect high-quality and discard low-quality ECG excerpts of about 93%, only misclassifying around 5% of clean AF segments as noisy ones. In addition, the method has also been able to deal with raw ECG recordings, without requiring signal preprocessing or feature extraction as previous stages. Consequently, it is particularly suitable for portable and wearable devices embedding, facilitating early detection of AF as well as other automatized diagnostic facilities by reliably providing high-quality ECG excerpts to further processing stages.

## 1. Introduction

Currently, atrial fibrillation (AF) is one of the major health challenges in the developed world, being the most common cardiac arrhythmia in clinical practice, roughly affecting 37.5 million people worldwide [1]. Moreover, since its prevalence is closely related to aging, it is expected to grow to epidemic proportions by the middle of this century [2,3]. Although AF is not life-threatening in itself, it reduces the patient’s quality of life and doubles the risk of death, compared with healthy individuals of the same age [4]. Indeed, this arrhythmia is the most common risk factor for ischemic stroke, because it provokes adverse hemodynamic alterations as well as rapid and irregular ventricular contractions [5,6]. However, pathophysiological mechanisms causing and maintaining AF are still not completely understood [7], thus making its therapy extremely challenging and often poorly effective [8]. To this respect, around one-third of hospitalizations for all cardiac disorders are directly associated with this arrhythmia [9].

In this context, early detection of AF is a priority to enable preventive treatments aimed at minimizing the arrhythmia burden, as well as the risk of its chronification [10,11]. However, that task is not easy, because about 90% of AF episodes are asymptomatic [12]. Moreover, most arrhythmic events in the initial stage of the disease often last a few seconds or minutes; their detection needs long-term continuous monitoring of a patient’s electrocardiogram (ECG) [13]. To this respect, previous works have proven that the longer the duration of monitoring, the greater the possibility of early identification of patients suffering from intermittent AF [14,15].

The most recent advances in portable and wearable medical devices may become highly useful in this premature screening of silent AF, because they are able to significantly increase the monitoring time window where the arrhythmia can be detected. Thus, fresh improvements in low-power embedded systems, communication protocols, and cloud computing technologies have allowed the development of numerous wearable systems with the ability for ECG monitoring over several weeks and even months, while the subject continues a normal active life [15,16,17,18]. However, these devices will usually work in highly dynamic and changing environments, thus providing ECG signals that are especially prone to be corrupted with different kinds of noises, such as motion artifacts, powerline interference, baseline wander, and high-frequency electromyography disturbances, among others [19].

Unfortunately, the presence of large levels of noise in ECG signals hampers their accurate interpretation, thus limiting diagnostic capabilities of any later system [20]. Moreover, strong artifacts and interferences have also been identified as responsible for most false alarms of AF occurrence in real-time ECG monitoring systems [21,22]. To palliate these problems, many algorithms have recently been proposed for ECG denoising [23]. However, their performance has been limited, since time and frequency components of most noises overlap with the ECG signal [24]. Furthermore, these methods can also introduce some artificial distortion in clean ECGs, thus leading to inaccurate diagnosis of several cardiac diseases [25,26]. Consequently, the automatic identification of non-contaminated, high-quality ECG excerpts is of paramount importance for portable and wearable ECG monitoring devices aimed at extracting reliable clinical information [20].

While a broad variety of techniques for ECG quality assessment have been proposed to date [20], most of them cannot deal with signals acquired via portable or wearable systems. On the one hand, many algorithms have been designed to simultaneously analyze the 12 leads found in the standard ECG, but portable and wearable systems often present a more reduced number of signals, commonly between one and three [15,16,17,18]. On the other hand, numerous methods raised to assess single-lead ECG quality are based on detecting fiducial points and morphological events in the signal, and then computing parameters such as mean RR interval, ratio of maximum to minimum RR interval, time consistency of PQRST waveforms, coherence of QRS complexes, etc. [20]. However, no accurate detection of fiducial points can be reached in very noisy ECG recordings, as well as in clean signals presenting time-varying waveforms or sharp T-waves [27,28]. These two aspects are common in long-term ECG recordings, and especially in those obtained from AF patients, since heartbeat irregularity is a major feature of the arrhythmic episodes [29]. Hence, it has been strongly recommended that quality assessment in single-lead ECG signals obtained through portable and wearable devices is not based on their morphological features [30].

On the other hand, although most previous ECG quality indices have reported promising results on recordings from healthy subjects, their performance on signals acquired from patients with different pathological cardiac conditions has shown to be fairly limited [31]. This is the case of many algorithms whose ability to discern between clean and noisy ECG excerpts has been significantly decreased when dealing with ECG recordings obtained from patients with atrial arrhythmias, including AF [23,30,32,33]. Hence, the present work aims at introducing a novel algorithm for quality assessment of single-lead ECG recordings acquired with portable and wearable devices from patients with intermittent AF.

The proposed method is based on deep learning techniques taking profit of the high learning capability of a convolutional neural network (CNN), which is able to extract the most relevant ECG features without delineation of its fiducial points, as well as without any other kind of manual or external intervention [34]. To jointly exploit time and frequency information in the single-lead ECG recording, the algorithm is fed with a 2-D image obtained by turning the raw signal into a scalogram through a continuous Wavelet transform (CWT). This approach has been successfully used in other ECG-based applications, such as classification of arrhythmias [35,36], automatic identification of AF [37,38], detection of diabetic subjects [39], detection of sleep apnea [40], estimation of systolic blood pressure [41], and biometric identification of individuals [42].

The remainder of this paper is organized as follows. Section 2 describes the databases analyzed to validate the proposed algorithm. Next, Section 3 outlines how ECG recordings are transformed into 2-D images using CWT and then inputted to the CNN for its training and testing. The numerous learning-testing cycles conducted for a robust validation of the method, along with the computed performance metrics, are also introduced in this section. Classification results between high- and low-quality ECG segments are next presented in Section 4 and later discussed in Section 5. Finally, Section 6 presents the concluding remarks of this study.

## 2. Databases

Three datasets were analyzed in the present study, i.e., the public PhysioNet/CinC Challenge 2017 database (PC2017DB) [43], the public Telehealth database (THDB) [44], and a proprietary database (PDB). They were chosen because ECG signals were acquired in diverse noisy and ever-changing environments, as well as using different portable or wearable recording systems. In this way, a broad variety of noises and ECG morphologies were then considered.

Briefly, the PC2017DB consists of 12,186 single-lead ECG recordings with a duration between 9 and 60 s [43]. They were acquired using an AliveCor™ device linked to a smartphone, at a sampling frequency of 300 Hz and 16 bits of resolution over a dynamic range of ±5 mV. Annotations from experts classifying the recordings into four groups, i.e., AF, normal sinus rhythm (NSR), other rhythms (OR), and noisy excerpts, are freely available. On the other hand, the THDB is formed by 300 single-lead ECG recordings with a length between 20 and 200 s, which were collected using a remote monitoring system (TeleMedCare™ Health Monitor) for 288 home-dwelling patients suffering from chronic obstructive pulmonary disease and/or congestive heart failure [44]. The signals were digitized at a sampling frequency of 500 Hz and 12 bits of resolution over a dynamic range of ±5 mV. Three experts independently revised the recordings to identify clean and noisy segments, these annotations being freely available. In the present study, 50 signals were discarded because they did not present any data. The remaining 250 ECGs were processed with an automatic algorithm to detect AF episodes within the clean ECG intervals [45] and later revised by an expert physician. Finally, the PDB comprises 36 single-lead ECG recordings lasting between 1 and 2 h from patients with intermittent AF which were recorded using a Nuubo™ textile wearable Holter. Acquisition of this dataset was approved by the Ethical Review Board of Hospital Universitario San Juan de Alicante (Protocol Number UGP–14–219, date 02/18/2015) and, after giving their consent, all subjects (20 women and 16 men, aged 52–68 years) were continuously monitored some weeks after a procedure of pulmonary vein isolation by catheter ablation. None of the selected patients presented underlying heart disease. The signals were recorded at a sampling frequency of 250 Hz and 12 bits of resolution over a dynamic range of ±5 mV. As before, an automatic algorithm was used to detect AF episodes [45], and two experts independently visualized the recordings to identify sufficiently clean and noisy intervals. ECG excerpts were labelled as noisy when either physician was unable to confidently distinguish all R-peaks.

To train and validate the proposed algorithm, all ECG signals from these datasets were divided into 5 s-length intervals and then grouped into two categories. Thus, segments from AF, NSR and OR episodes constituted the group of high-quality ECGs, and noisy segments formed the group of low-quality ECGs. Table 1 shows the total number of ECG excerpts for each group analyzed from the three datasets. As can be seen, the PC2017DB and the PDB were notably unbalanced, because they presented a significantly larger percentage of high-quality ECG segments (97.58 and 79.21%, respectively) than low-quality ones (2.42 and 20.79%, respectively). Nonetheless, more than 96,600 5 s-length ECG segments were analyzed in total.

## 3. Methodology

The proposed algorithm to discern between high- and low-quality ECG intervals consists of two steps. Firstly, every ECG excerpt is turned into a 2-D image, and secondly a properly trained CNN obtains its probability of belonging to one out of the two classes, thus providing a final classification outcome. Additional details are next described.

### 3.1. Continuous Wavelet Transform

To transform each 5 s-length ECG segment into a time-frequency representation, CWT was used. Compared to other time-frequency transformations, like short-time Fourier transform, CWT presents a superior ability to accurately detect local, transient and intermittent aperiodicities in non-stationary signals [46], such as ECG recordings. Moreover, algorithms based on CWT have also performed better in pattern recognition and classification problems than others based on conventional cosine and Fourier transforms [47]. In brief, CWT decomposes a signal at different time scales, each one representing a certain frequency range in the time-frequency plane [48]. More precisely, the original signal is correlated with scaled and shifted versions of a wavelet function, which is named mother wavelet, consisting of a smooth and quickly vanishing oscillation with good localization both in frequency and time [48]. From a mathematical point of view, CWT of a signal x(t) is defined as [49]

(1) $$\begin{array}{cc} CWT(a,b) & =\frac{1}{\sqrt{a}}\int_{-\infty }^{+\infty }x\left(t\right)\psi^{*}\left(\frac{t-b}{a}\right)dt, \end{array}$$

where a,b∈ℜ,a≠0 are the scaling and shifting parameters, respectively, ψ(t) is the mother wavelet, and * denotes the complex conjugate operator. This transformation results in a 2-D matrix composed of wavelet coefficients located according to their scale and position. Visual representation of the absolute value of these wavelet coefficients using a proper colormap is known as wavelet scalogram, and has been widely used for making interpretation of this time-frequency transformation easier [50].

**Figure 1:**
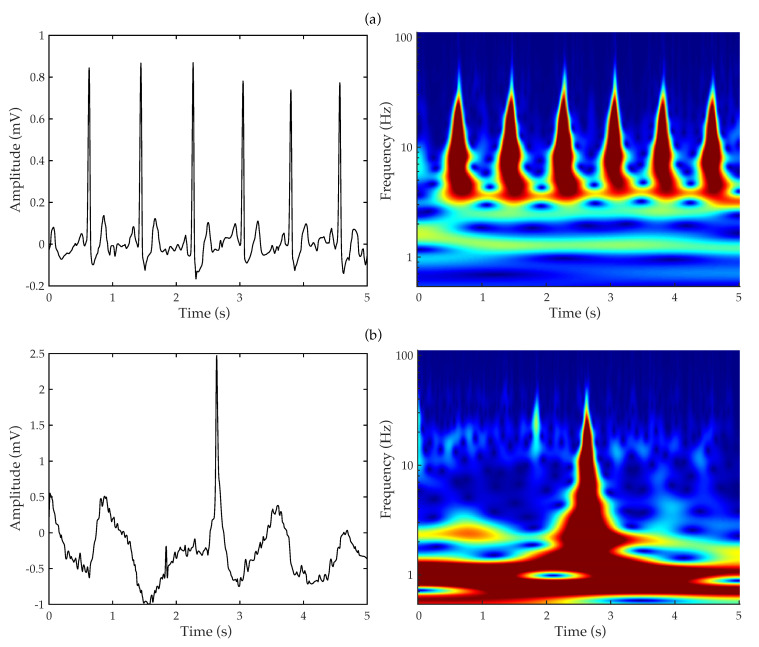
Example of typical 5 s-length ECG intervals from the (**a**) high- and (**b**) low-quality groups, along with their corresponding scalograms.

The resulting scalogram for a specific signal strongly depends on the chosen number of wavelet scales, the used mother wavelet function, and the selected colormap. In the present study, a Morlet function, composed of a complex exponential function multiplied by a Gaussian window, was used as mother wavelet. This function has been broadly used in other ECG-based applications [36,38,40,42], because it shows equal variance in time and frequency [51]. Moreover, the number of scales was determined by the energy spread of the wavelet in time and frequency when 48 voices per octave was used. Finally, a Jet colormap with 128 colors was used to obtain the final scalogram. As an example, Figure 1 shows two common 5 s-length ECG segments from high- and low-quality groups, along with their corresponding wavelet scalograms. Whereas the high-quality ECG excerpt exhibits a scalogram with a clear repetitive pattern, the low-quality one displays an arbitrary image owing to the presence of a strong motion artifact and a protruding baseline.

### 3.2. Convolutional Neural Network

In the last decade, ECG classification has been the focus of numerous studies based on machine learning techniques. Indeed, a variety of algorithms, including linear and quadratic discriminant analyses, support vector machines and artificial neural networks, have been used for heartbeat classification, abnormal heart rhythm identification, and even ECG quality assessment [20,52]. However, these methods need to be trained with parameters explicitly extracted from the ECG, and therefore its fiducial points and waveforms have to be firstly detected and delineated [52]. Recently introduced techniques based on deep learning, such as CNNs, avoid this tricky step, since feature extraction is an intrinsic part of their learning [53]. Moreover, they have also shown to be powerful and highly accurate in many different applications [54], including those dealing with ECG recordings [53]. Another interesting advantage of these algorithms is their ability to successfully work with raw signals, without demanding any kind of preprocessing [55].

**Figure 2:**
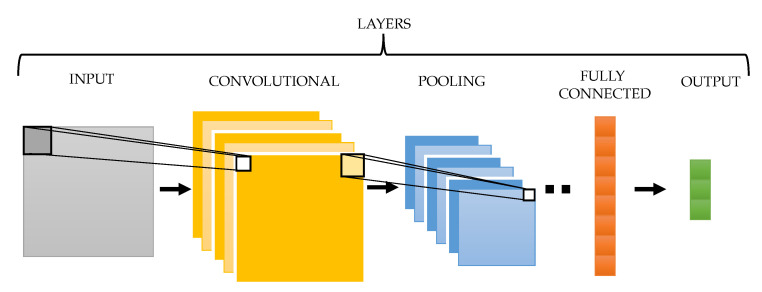
Structure of the usual pipeline of a general 2-D CNN architecture.

The typical structure of a 2-D CNN is displayed in Figure 2. As can be seen, this kind of network consists of a set of different layers that operate in a sequential and/or parallel way. More precisely, after receiving a 2-D image as input, the method presents a variable number of different layers. The convolutional layer extracts local features from the input image by its convolution with different filters [56]. This layer is generally followed by a pooling one, which combines similar features to make the model simpler and more robust to noise and input deformations. In this way, the resulting features represent the original image from different angles in an enhanced manner. As the number of these layers increases, input image representation becomes more and more abstract [54]. Finally, the fully connected layer converts the 2-D feature maps into a 1-D feature vector for further representation. In every CNN the last layer will be a fully connected one, which will provide the information needed to compute the probability distribution of belonging to each output category or class [54]. Apart from these layers, other mathematical functions, such as rectified linear units (ReLU), data normalizations or dropout regularizations, can be included in intermediate points of the network to enhance its generalization capability [54].

It is interesting to note that CNNs are rarely trained from scratch [57]. In fact, this is extremely challenging for ECG-based applications, given the limited availability of large-scale datasets annotated by expert cardiologists, as well as the large time required to collect and label ECG recordings in some clinical scenarios [53]. Instead, a common practice is to take a pre-trained CNN on a non-specific large dataset as a starting point and then developing its fine-tuning on the problem in hand [57]. This approach is known as *transfer learning*, and has been applied in the present study. More precisely, the well-known CNN AlexNet [58] was adapted and specifically re-trained to discern between high- and low-quality ECG segments. This network has been widely used in diverse classification problems, since it has been pre-trained with more than 1.2 million of images to discern among 1000 classes [59,60,61]. The re-training process of the CNN was developed through a stochastic gradient descent algorithm with a momentum of 0.9 and a learning rate of 0.0001.

**Figure 3:**
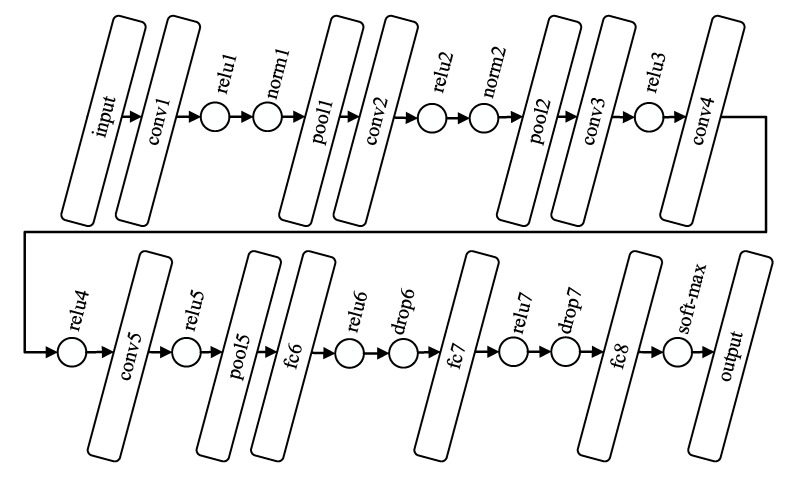
Illustration of the layer-based architecture of AlexNet [58].

The original architecture of AlexNet is displayed in Figure 3. As can be seen, the algorithm is composed by eight layers with ability to learn, i.e., five convolutional and three fully connected ones [58]. After these layers, common ReLU activation functions (*relu1*, *relu2*, *relu3*, *relu4*, *relu5*, *relu6*, and *relu7*) are found. The first convolutional layer (*conv1*) filters the 227 × 227 × 3 fixed-size input image with 96 kernels of size 11 × 11 × 3. Then, the output is normalized (*norm1*) and pooled (*pool1*) before being inputted to the second convolutional layer (*conv2*), which filters the feature space with 256 kernels of size 5 × 5 × 48. Next, the feature space is again normalized (*norm2*) and pooled (*pool2*) before reaching the third convolutional layer (*conv3*), which filters the feature space with 384 kernels of size 3 × 3 × 256. The fourth and fifth convolutional layers (*conv4* and *conv5*) present 384 and 256 kernels, respectively, both with the same size of 3 × 3 × 192. After a pooling layer (*pool5*), three fully connected ones (*fc6*, *fc7*, and *fc8*) are connected in cascade with two intermediate drop-out regularizations (*drop6* and *drop7*). Finally, the last fully connected layer feeds a 1000-way *soft-max* function, which computes the probability distribution of belonging to 1000 output classes. In the present study, this final function was modified to produce a two-class output.

### 3.3. Experimental Setup and Performance Assessment

As previously described in Section 2, the PC2017DB and the PDB were notably unbalanced, both containing a much larger number of high-quality ECG segments than low-quality ones (see Table 1). To avoid the effect of this imbalance on classification, the proposed method was exposed to several validation cycles using different subsets of ECG segments from each database. More precisely, 40 iterations were run for the PC2017DB, such that in each one all 1168 low-quality ECG intervals were maintained and other 1168 samples were randomly selected from the high-quality group. It should be noted that this last subset was stratified by selecting 468 NSR segments, 340 AF intervals and 330 OR excerpts. Making use of the same approach, 19 validation cycles were conducted from the PDB. Thus, two subset of 1200 samples were randomly selected from high- and low-quality groups, respectively. As before, the high-quality subset was evenly composed of 600 NSR segments and 600 AF intervals. Regarding the THDB, only one validation cycle was completed, because the number of high and low-quality ECG segments was well-balanced in this case. Finally, 40 cycles were also conducted by considering jointly samples from the three databases. Thus, in each iteration both high- and low-quality subsets were comprised of 2500 ECG excerpts, 1000 randomly selected from the PC2017DB, 500 from the THDB, and 1000 from the PDB. As in previous experiments, the high-quality group was stratified by considering a similar number of ECG intervals from NSR, AF, and OR episodes.

To obtain a robust classification outcome in each one of these 100 validation cycles, a holdout approach with a stratified 80/20 split (80% for learning and 20% for testing) was run five times. Hence, the proposed algorithm was trained and tested 200 times from the PC2017DB, 95 from the PDB, five from the THDB, and 200 from samples randomly chosen from all datasets. Classification results for each iteration were assessed in terms of sensitivity (Se), specificity (Sp) and accuracy (Acc), and then mean, standard deviation (std), maximum, and minimum values of these performance metrics were computed for each database. Whereas Se was defined as the rate of correctly classified high-quality ECG segments, specificity (Sp) was estimated as the percentage of properly identified low-quality intervals. The total number of rightly detected ECG excerpts was finally the Acc. From a mathematical point of view, these performance metrics were computed as

(2) $$Se=\frac{TP}{TP+FN},$$

(3) $$Sp=\frac{TN}{TN+FP},\;\mathrm{and}$$

(4) $$Acc=\frac{TN+TP}{TN+TP+FN+FP},$$

where TP was the number of correctly identified high-quality ECG segments, TN the amount of correctly classified low-quality segments, FP the number of low-quality segments improperly classified as high-quality ones, and FN the amount of high-quality intervals wrongly identified as low-quality ones. Finally, the rates of correctly classified NSR ($R_{NSR}$), AF ($R_{AF}$) and OR ($R_{OR}$) intervals within the high-quality group were also computed and averaged for all validation cycles.

Finally, the method most commonly used as a reference in previous works, such as in [31,32,62,63,64], has also been implemented and validated with the described approach. This algorithm was proposed by Clifford et al. [65] and is based on combining four ECG-based parameters, such as, the percentage of R-peaks identified by two published detectors, the relative power in the QRS complex, the fourth moment (i.e., kurtosis) of the signal, and the relative power in the baseline, through a support vector machine (SVM) classifier with a Gaussian kernel and parameters C=25 and γ=1. More details can be found in [65].

## 4. Results

Classification outcomes obtained by the proposed method for all conducted experiments are presented in Table 2. As can be seen, the algorithm reported a slightly poorer performance on the PC2017DB than on the remaining datasets. Nonetheless, in this case mean values larger than 85% were still observed for all performance indices, which also exhibited limited dispersion among validation cycles. To this respect, values of std lower than 3.6% and differences between maximum and minimum data ranging from 5 to 14% were reported. Of note is also that Sp and Se were well-balanced, although Sp was marginally larger than Se (i.e., 91 vs. 87%). Within the high-quality ECG group, the classification rates of AF and OR intervals were mostly identical and about 86% in average, but the proportion of correctly identified NSR segments was a little bit higher, i.e., about 90%.

Compared to the PC2017DB, mean Acc was only increased by 2.5% when ECG signals from the THDB were analyzed. However, a trend reversal in values of Se and Sp was noticed. Thus, Se was 10% greater than Sp, both values being about 95 and 85% in average, respectively. According to this increase in Se, the classification rates of NSR and AF segments within the high-quality ECG group also presented values around 95%. Both were well-balanced, even though a much larger number of NSR intervals than AF ones were analyzed in this case. Regarding the PDB, very similar outcomes were also observed, but average values of Sp and Acc significantly increased to almost 93 and 95%, respectively. It is also worth noting that the percentage of correctly detected AF excerpts was maintained about 94%, but a notable increase in the number of properly identified NSR segments was reported, reaching a rate larger than 99.5%. For both databases, no great dispersion among validation iterations was noticed in all performance metrics. Indeed, values of std roughly ranged from 1 to 5.5%, and differences between maximum and minimum data from 2 to 13%.

When ECG intervals from all databases were combined, the classification results obtained by the proposed method were midway between those previously described. Thus, mean values of Se, Sp, and Acc were about 94, 91 and 92%, respectively. Moreover, within the high-quality ECG group, the proportions of correctly identified NSR and AF episodes were well-rounded with values about 92%. As before, no strong variations were noticed among learning-testing cycles, because values of std roughly remained between 1 and 3.5%, and differences between maximum to minimum data oscillated from 2 to 7% for all performance metrics.

Finally, the classification results obtained by the Clifford et al.’s algorithm [65] are presented in Table 3. As can be seen, compared to those reported by the proposed algorithm in Table 2, mean values of all performance metrics were between 3 and 19% lower for all conducted experiments. Moreover, considering the global performance in terms of Acc, this method incorrectly classified around 8, 15, 7 and 8% more the total number of ECG excerpts for the four tested databases, respectively. Similarly, within the high-quality ECG group, the method’s ability to properly identify NSR and AF intervals was also reduced by about 3–13% in comparison with the proposed algorithm. Regarding the results obtained for all validation iterations, in most cases no great differences were noticed between minimum and maximum data, with values of std mainly remaining between 1 and 3%.

## 5. Discussion

To the best of our knowledge, the present work has introduced for the first time an algorithm to detect high-quality segments in single-lead ECG recordings obtained from patients with intermittent AF. The method has been broadly trained and validated on a variety of signals acquired with several portable or wearable devices. By cumulating the total number of recordings tested in all the iterations and database combinations, more than 320.000 ECGs have been analyzed. In addition, the ECG signals in diverse databases reflected heart electrical activity from different body positions. Whereas in the PC2017DB and the THDB a lead I equivalent ECG was captured by electrodes located at each hand of the patient [43,44], in the PDB a non-standard lead was acquired from the patient’s thorax [15]. Similarly, the ECG recordings in each database also presented diverse kinds and levels of noises, because they were obtained from different environments and making use of different approaches. While the recordings in the THDB were only captured from an in-home tele-health context [44], those in the PC2017DB and the PDB were obtained from more dynamic environments, including out-of-home scenarios [43]. Moreover, in the PC2017DB dataset, the signals were acquired under the subject’s request and then the probability to capture noise and motion artifacts was reduced. Contrarily, in the THDB and the PDB the ECGs were continuously recorded while the subjects continued a normal life, this context being more sensitive to perturbations and interferences.

Despite these differences among ECG signals, similar mean values of Acc were obtained for the three databases, as well as for the experiment in which samples from them were combined. In fact, only differences lower than 6% were noticed in the four analyses (see Table 2). No significant variations among mean values of Se and Sp were also seen, but a change in the method’s performance for both ECG groups and each database was observed. Thus, whereas a larger proportion of high-quality intervals were correctly detected in the THDB and the PDB, a greater rate of low-quality excerpts were properly classified in the PC2017DB. Because, in addition to NSR and AF episodes, OR were considered in this last dataset, the wider variety of ECG morphologies considered in the high-quality group could explain that result. As in most clinical applications [66], well-balanced values of Se and Sp are also desirable in this case, because both the risk of misdiagnosis by interpreting noisy signals and the loss of clinical information by discarding clean ECG excerpts would be equally reduced. However, maximizing detection of low-quality ECG segments at the cost of slightly increasing the rate of false positives could not be troublesome in the context of wearable systems, since they usually record every day many hours of clean ECG signals, so that high-quality intervals could be easily analyzed.

The broader variety of ECG morphologies in the PC2017DB could also justify the slightly poorer global performance of the method on this database than on the remaining ones. Nonetheless, the proposed algorithm reached values of Se, Sp, and Acc near o higher than 90% for all conducted experiments. In general term, these outcomes are comparable to those reported by most previous works dealing with quality assessment of single-lead ECG recordings, which are summarized in Table 4. However, comparison of these methods with the proposed one should be established with caution due to two major reasons. On the one hand, whereas a robust validation approach was developed in the present work by conducting 500 learning-testing iterations and involving more than 96,000 5-s-length ECG excerpts from three databases, a limited number of samples was used in some previous studies. For instance, to obtain the large classification rates presented in the table, Satija and colleagues selected only 7000 and 9818 10 s-length ECG intervals for training and testing the algorithms introduced in the works [30] and [32], respectively. Moreover, although the dataset analyzed in [32] was notably unbalanced, presenting 1404 high-quality ECG intervals and 8414 low-quality ones, the authors did not discussed how overrepresentation of noisy signals could have biased their classification results. In a similar way, Zhao & Zhang [63] only choosed 300 30 s-length ECG intervals (150 for each group) from the PC2017DB for training and validation of their technique. In both cases, a full inclusion of all recordings available in the database would have provided a more fair comparison of results.

On the other hand, it should also be noted that results presented in Table 4 were mainly obtained from NSR recordings. In fact, most previous works analyzed the public database proposed for the PhysioNet/CinC Challenge 2011, which contains 1500 10 s-length ECG excerpts recorded from healthy subjects [67]. However, this validation context is less challenging than considering jointly NSR and AF segments in the high-quality group, such as in the present work, because a more limited variety of ECG morphologies are studied [31]. Moreover, some authors have also found that several kinds of noises exhibit time and spectral characteristics very similar to AF and other atrial arrhythmias, thus significantly impacting on ECG quality classification when patients suffering from these pathologies are considered [22,31]. To this respect, Table 3 shows how the performance of the Clifford et al.’s algorithm was in general terms reduced by about 10–20% regarding the results provided in the original work [65], as well as regarding those presented by the proposed CNN-based algorithm in Table 2. In the same line, the performance of the Behar et al.’s algorithm was also decreased by 10% when ECG signals from patients with several atrial arrhythmias (including AF) were studied [31]. Likewise, Satija and colleagues implemented several algorithms previously proposed in the literature and noticed that most of them only reached values of Acc between 50 and 70% when validated with ECG recordings from patients with AF and other atrial arrhythmias [20,32]. In contrast to these outcomes, the proposed algorithm has provided a promising ability to discern between high-quality AF and noisy excerpts, because mean values of $R_{AF}$ ranged between 86 and 96% for all conducted experiments (see Table 2).

With regard to the previous works summarized in Table 4, another advantage of the proposed algorithm is its ability to directly deal with ECG signals provided by portable and wearable systems, without demanding any kind of preprocessing stage (such as denoising or downsampling), tedious ECG-based or R-peak-based feature computation, manual or automatic feature selection, and any other kind of manual or external intervention. This way, the algorithm is particularly suitable for wearable systems, thus ensuring that only ECG intervals with sufficient quality are feeding later algorithms for further analysis. The processing of only noise-free ECG intervals would avoid most of confounding factors in ECG interpretation and then, more reliable analyses, more accurate diagnoses, and smaller rates of false alarms of AF and other atrial arrhythmias could be obtained in continuous long-term ECG monitoring [20,31]. This is especially relevant in the screening of AF, because episodes are too short in the initial stages of the disease [13] and reliable automatic analyses could be helpful in minimizing or even removing later visual, time-consuming inspection of suspicious events, as well as in avoiding desensitization of the clinical staff responsible for this task [70]. Natural progression of the arrhythmia could then be more successfully and early prevented, hence eluding chronic stages where management of the disease is more complicated and treatments are poorly effective [71].

Nonetheless, the aforementioned characteristics can also be found in other CNN-based methods dealing with quality assessment of single-lead ECG recordings. The most relevant algorithms in this respect are summarized in Table 5. So far, a few CNNs with different architectures have only been proposed to discern between ECG intervals with different levels of quality. For instance, Zhou et al. [72] proposed a very basic 1-D CNN to classify between high- and low-quality intervals. Although a good classification result was reported, the authors only selected about 5000 out of 18,000 ECG excerpts available from the PhysioNet/CinC Challenge 2011 database, thus ignoring AF and other atrial arrhythmias. Yoon et al. [23] introduced four models based on combining two similar 1-D CNNs to work in parallel, such that one network was fed with the original ECG and another one with its spectral distribution. Discerning between 2700 ECG segments divided into two groups (acceptable and non-acceptable for further diagnosis), the best model achieved an accuracy about 88%. However, this good performance drastically fell to 50% when AF recordings were considered in the study. A similar structure of two CNNs working in parallel was also proposed by Zhang et al. [33] to identify three levels of noise (low, mild, and severe). The first network was one-dimensional and received original ECG as input, whereas the second one was two-dimensional and then fed with the ECG wavelet scalogram. The method was trained and validated with ECG recordings from patients with a variety of cardiac pathologies (including AF), and a promising discriminant ability about 92% was obtained in global terms. However, when recordings alternating AF and NSR were separately analyzed, its performance was significantly reduced by 15%. Finally, Zhao et al. [73] designed a 2-D CNN with 13 layers to discern among high-quality ECG excerpts, clinically useful ECG segments with poor quality, and clinically useless ECG intervals. After training and testing the algorithm with only 1000 10 s-length ECG segments from healthy subjects, an accuracy about 86% was achieved.

Although the results just described for these methods were promising, the CNN-based technique proposed in the present study has still reported a notably better performance, especially in the context of AF. The fact that in these previous works customized CNNs were trained from scratch using limited datasets could explain this finding. Hence, as in other ECG-based applications [57], taking advantage of pre-trained CNNs seems to be an efficient alternative to improve quality classification of single-lead recordings, at least when a broad variety of ECG morphologies from several cardiac conditions are considered and no tens of thousands of samples from each one are available.

Finally, some limitations of this study deserve attention. Firstly, the main drawback of every CNN-based method is the difficulty to understand the rationale behind its results. This kind of algorithm is unable to provide explanations about the pathophysiological basis of its classification outcomes, keeping every functional dependency between inputs and outputs hidden [53]. On the other hand, OR episodes were not included in the THDB and the PDB, because sufficiently representative samples were not found during preliminary ECG recordings inspection and classification. Nonetheless, beyond a few episodes of atrial flutter and ventricular bigeminy or trigeminy, no other abnormal rhythms are often found in long-term ECG recordings obtained from patients with intermittent AF [74], and therefore the present study considered a totally realistic scenario.

**Figure 4:**
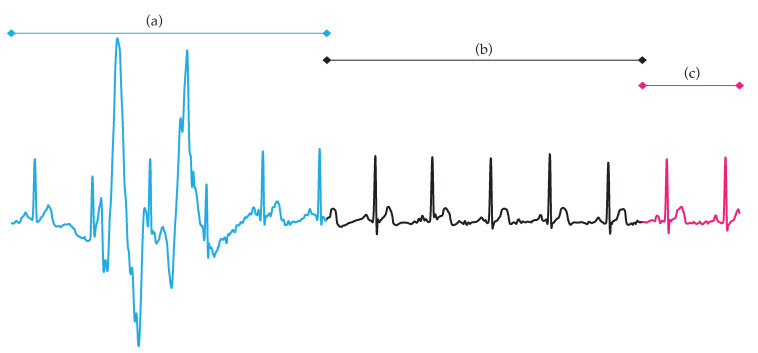
A noisy ECG recording from the PC2017DB segmented into 5 s-length excerpts. The first ECG segment (a) was labelled as low-quality and coherently presented a high level of noise. The second ECG interval (b) was labelled as low-quality but exhibited sufficient quality for further analysis. The last ECG excerpt (c) was discarded because its length was shorter than 5 seconds.

Furthermore, inconsistency in labels of some ECG excerpts could exist, because the three analyzed databases were annotated by different experts and quality labelling is a subjective task [31]. Also, segmentation of ECG signals from the PC2017DB into 5 s-length intervals generated some twrong labels. Since a single label was assigned to each ECG signal (regardless of its duration) and highly localized noise was noticed in some cases, a few ECG segments extracted from noisy recordings were acceptably clean. An example of this is shown in Figure 4, where the second 5 s-length excerpt should have been labelled as high-quality, instead of low-quality. Hence, relabelling of all ECG signals with clear and consistent rules, such as in [64], could have improvted the obtained classification results. Nonetheless, this analysis will be conducted in further works. An additional study identifying several quality levels will also be tackled in the future, because ECG intervals corrupted with moderate noise could be confidently used for some kinds of diagnoses, e.g., for those based on heart rate variability [62].

## 6. Conclusions

A novel deep learning-based algorithm able to reliably identify high-quality signal intervals within the challenging environment of single-lead ECG recordings from wearable devices mainly alternating NSR and AF episodes has been presented. After exhaustive training and validation on several and diverse datasets, the method has proven a significantly better performance than previous techniques. Moreover, because it is based on exploiting time and frequency information in the ECG through a pre-trained CNN, no preprocessing and feature extraction stages were required, thus making its use particularly suitable for wearable devices. This way, further processing of only high-quality raw ECG excerpts is guaranteed for later stages, so that accurate early detection of AF, as well as other reliable automatized diagnoses, could be easily achieved from very long-term continuous monitoring in portable and wearable devices.

## Figures and Tables

**Table 1:**
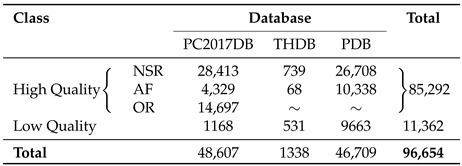
Total number of 5 s-length ECG excerpts for high- and low-quality groups analyzed from each database.

**Table 2:** Summary of classification results obtained by the proposed algorithm to discern between high- and low-quality ECG segments for all conducted experiments in the datasets.

Database	Value	Se (%)	Sp (%)	Acc (%)	$R_{NSR}$ (%)	$R_{AF}$ (%)	$R_{OR}$ (%)
**PC2017DB**	Mean	86.91	91.00	88.95	89.28	85.92	85.69
	Std	2.64	2.66	1.03	2.80	3.18	3.58
	Maximum	93.10	96.40	91.80	95.55	91.61	93.84
	Minimum	81.70	85.00	87.05	82.33	77.91	79.28
**THDB**	Mean	95.49	85.00	91.42	96.50	94.04	—
	Std	2.71	4.74	1.08	5.59	2.85	—
	Maximum	98.78	88.46	92.91	100	99.31	—
	Minimum	92.68	76.92	90.30	87.50	92.92	—
**PDB**	Mean	97.17	92.42	94.79	99.58	93.86	—
	Std	1.57	3.14	1.41	0.94	2.75	—
	Maximum	98.75	96.25	96.88	100	97.09	—
	Minimum	94.58	88.75	93.33	97.90	89.69	—
**Samples from** **previous datasets**	Mean	94.42	90.61	92.51	92.87	92.05	—
	Std	3.44	3.45	1.08	1.35	2.23	—
	Maximum	96.65	92.29	91.43	97.51	94.59	—
	Minimum	89.08	86.08	89.40	94.21	88.56	—

**Table 3:** Summary of classification results obtained by the Clifford et al.’s work [65] to discern between high- and low-quality ECG segments for all conducted experiments in the datasets.

Database	Value	Se (%)	Sp (%)	Acc (%)	$R_{NSR}$ (%)	$R_{AF}$ (%)
**PC2017DB**	Mean	80.84	81.17	81.01	79.97	81.73
	Std	1.72	1.61	1.08	2.10	2.29
	Maximum	84.10	86.20	83.70	83.80	86.30
	Minimum	77.10	78.50	78.80	75.82	76.48
**THDB**	Mean	84.09	66.03	76.92	83.68	89.10
	Std	4.20	5.25	3.06	4.46	8.85
	Maximum	90.68	72.64	82.02	90.54	100
	Minimum	79.50	59.43	73.78	78.37	76.92
**PDB**	Mean	87.97	86.94	87.45	94.51	81.08
	Std	1.08	1.61	0.82	0.85	1.81
	Maximum	89.90	90.40	89.50	96.20	84.11
	Minimum	85.60	83.90	85.55	92.41	76.93
**Samples from** **previous datasets**	Mean	84.49	85.43	84.43	90.24	80.11
	Std	0.79	1.50	0.96	1.14	1.26
	Maximum	85.22	86.50	85.54	91.80	81.85
	Minimum	83.29	82.86	83.08	88.70	78.92

**Table 4:** Main features and results achieved by previous non-CNN-based algorithms dealing with quality assessment of single-lead ECG recordings.

Work	Methodology	Classes	Main Results
Behar et al. [31]	Seven ECG-based indices	High- and	Acc = 98.4%
	combined with a SVM classifier	low-quality ECGs	
Moeyersons et al. [64]	Descriptive features from autocorrelation	High- and	Se = 97.7%
	function combined with a RUSBoost classifier	low-quality ECGs	Sp = 94.7%
Clifford et al. [65]	Four ECG-based indices	High- and	Se = 95.8%
	combined with a	low-quality	Sp = 97.2%
	SVM classifier	ECGs	Acc = 96.5%
Orphanidou et al. [68]	Analysis detected R-peaks and	High- and	Se = 97%
	correlation QRS complexes with a template	low-quality ECGs	Sp = 94%
Hayn et al. [69]	Multiple QRS-based parameters	High- and	Acc = 91.3%
	combined with rules	low-quality ECGs	
Zhao & Zhang [63]	Multiple R-peak-based parameters	High- and	Se = 97.33%
	combined with rules and	low-quality	Sp = 88.67%
	Fuzzy synthesis	ECGs	Acc = 92.57%
Satija et al. [30]	Parameters extracted from wavelet	High- and	Se = 99.53%
	decomposition of the ECG and	low-quality	Sp = 98.95%
	combined with rules	ECGs	Acc = 99.16%
Satija et al. [32]	Parameters extracted from empirical mode	High- and	Se = 98.56%
	decomposition of the ECG and	low-quality	Sp = 99.12%
	combined with rules	ECGs	Acc = 98.90%

**Table 5:** Main features and results achieved by previous CNN-based algorithms dealing with quality assessment of single-lead ECG recordings.

Work	Methodology	Classes	Main Results
Zhou et al. [72]	A 1-D CNN fed with the ECG	High- and	Se = 95.5%
		low-quality	Sp = 91.3%
		ECGs	Acc = 94.3%
Yoon et al. [23]	Two 1-D CNNs working	High-	Se = 89%
	in parallel with	and	Sp = 88%
	ECG and its	low-quality	Acc = 88%
	spectral distribution	ECGs	$R_{AF}$ = 50%
Zhang et al. [33]	Two stages with two CNNs	ECGs with three	Acc = 91.8%
	(1D and 2D) working in parallel	levels of noise	$R_{AF}$ = 75–83%
Zhao et al. [73]	A 2-D CNN fed with wavelet	ECGs with three	Acc= 86.3%
	scalogram of the ECG	levels of noise	
